# Analysis of the level of clinical skills of physician assistants tested with simulated intensive care patients

**DOI:** 10.1111/jep.12937

**Published:** 2018-05-28

**Authors:** Anneke J.A.H. van Vught, Geert T.W.J. van den Brink, Murielle G.E.C. Hilkens, Jos A.H. van Oers

**Affiliations:** ^1^ Faculty of Health, Behavior and Society HAN University of Applied Sciences Postbus 6960 Nijmegen HG 6503 Netherlands; ^2^ Department of Intensive Care Radboud University Medical Center Postbus 9101 Nijmegen HB 6500 Netherlands; ^3^ Department of Intensive care Elisabeth‐TweeSteden Ziekenhuis Postbus 90151 5000 LC Tilburg Netherlands

**Keywords:** clinical skills, critical care, ICU, intensive care unit, physician assistant, substitution of care

## Abstract

**Rationale, aims, and objectives:**

Since adequate staffing in intensive care units (ICUs) is an increasing problem worldwide, we investigated whether physician assistants (PAs) are able to substitute medical residents (MR) in ICUs with at least the same quality of clinical skills. In this study, we analysed the level of clinical skills of PAs in direct comparison with those who traditionally performed these tasks, ie, MR with 6 to 24 months of work experience in the ICU.

**Method:**

Physician assistants and MRs in the ICUs were observed on their clinical skills by means of a simulated ICU comprising 2 scenarios on a human patient simulator with typical ICU cases. The level of clinical skills of PAs and MRs was videotaped and scored with predefined checklists by 2 independent intensivists per scenario. Percentage of the total score was calculated, and means were compared by Student *t* test.

**Results:**

A total of 11 PAs and 10 MRs participated in the study. Physician assistants and MRs scored equal (PA 66% ± 13% vs MR 68% ± 9%, *P* = .86) on their clinical performance in the simulated ICU setting.

**Conclusion:**

This study showed equal performance of PAs and MRs on clinical skills in the simulated ICU setting.

## 1 INTRODUCTION

Substitution of care from medical doctors (MDs) to physician assistants (PAs) is increasingly being adopted as a strategy to augment staffing and promote continuity and quality care in hospitals, including the intensive care unit (ICU).^1^, ^2^, ^3^, ^4^ Use of PAs in multidisciplinary staffing has been identified as a solution for meeting the shortage of medical doctors on the ward, health care costs, improving continuity of care and doctor's workload.

Physician assistants are licensed to practise medicine in defined domains, in collaboration with MDs but with a substantial degree of professional autonomy. Physician assistants obtain medical history, perform physical examinations, request and interpret additional testing, obtain medical diagnoses and treatment procedures, and prescribe medication. They also perform endoscopies, catheterizations, elective cardioversion, and minor surgery.^1^, ^5^, ^6^, ^7^ Physician assistant programmes lead to the award of a master's degree and are based on the medical model. Physician assistant programmes require health care experience as a health care professional on bachelor degree of at least 2 years. Dutch PA programmes incorporate a dual work‐education model, which means that students are employed as a PA in training within a particular medical specialty while enrolled in the master's PA programme.^6^ Dutch students follow a two and a half years training programme and spend nearly one‐third of their training time in one specialty, one‐third in various clinical specialties (clinical rotation), and one‐third in the classroom.^6^

As of 2018, there are approximately 1200 clinically active PAs in the Netherlands. Growth is expected around 200 per year at least to 2025. The number of PAs educated and trained to work in ICUs is approximately 40 (personal communication, President Netherlands Association of Physician Assistants—November 2017). In intensive and acute care units, general roles and responsibilities of PAs include assessing patients, obtaining patients' history and doing physical examinations, making rounds with the multidisciplinary team, performing invasive procedures (eg, suturing, placing central, and arterial catheters), and assisting in surgery under the supervision of an intensivist. Other roles include serving as first responders for institutional rapid response teams and cardiac arrest teams, doing evaluation and triage for patients outside the ICU, acting as a preceptor for medical and nursing students, providing support and education to the clinical nursing staff, and communicating with patients and patients' families.^4^, ^8^

International research, mainly from North America has shown that employment of PAs can improve efficiency, which is defined here as performing the same tasks as professionals trained in a different track with lower economic costs and educational level.^1^, ^2^, ^8^, ^9^ Of high importance is the quality of clinical care. Several studies have examined the performance of PAs and suggest that PAs can provide high‐quality care in a large range of medical disciplines. A systematic review by Kleinpell et al^8^ showed that patients' outcomes in the ICU were similar or better when care was delivered by an ICU team with PAs compared with care delivered by an ICU team without PAs; however, the evidence of the studies included was low. Quality of care is an outcome that is based on many more factors than the performance of an individual professional, for instance, patient‐provider ratio, collaboration within the team and the performance of the intensivists. Insight in the clinical competences of PAs in comparison with the level of clinical competences of medical residents (MR) who traditionally performed the substituted tasks is of pivotal value for quality and safety of care.

Since PAs take over tasks from MRs, the qualities to that tasks should not differ from that of MRs. Previous research showed no difference in the level of clinical skills of PAs compared with MRs in the specialty in which they are employed.^10^, ^11^ A study performed in a simulated preoperative anaesthesiology outpatient clinic showed no difference in clinical competencies (ie, history taking, physical exam, communication, and report) of PAs and second‐year anaesthesiology residents.^10^ Another study that investigated clinical skills of PAs and MRs at the surgery department showed equal performance of PA students and medical residents.^11^ However, both studies evaluated the level of skills that are specific to the particular specialty, not the ICU.

Since adequate staffing in ICUs is an increasing problem worldwide as well as in the Netherlands,^3^ we investigated whether PAs are able to substitute medical residents in ICUs with at least the same quality of clinical skills. In this study, we analysed the level of clinical skills of PAs in direct comparison with those who traditionally performed these tasks, ie, MRs with 6 to 24 months of work experience in the ICU.

The outcome of the study gives insight to the quality of care provided by PAs in ICUs and may be helpful for hospital administrators and medical leaders in considering the utilization of PAs in this setting.

## 2 METHODS

### 2.1 Population and design

In this observational study, the level of clinical skills of PAs and medical residents working in the ICU was assessed by using the human patient simulator (HPS). In the Netherlands, PAs are substitutes for MRs with 6 to 24 months of work experience in the ICU. These MRs are recently graduated medical doctors and not yet in training to become specialists. The goal was to include 11 PAs and 11 MRs due to costs.

From 2015 to 2016, PAs were invited through mailings of the authors' network and the professional association to participate in this study. Medical residents were invited through intensivists. Two intensivists, one from a teaching hospital and one from a university hospital, invited all MRs with 6 to 24 months of work experience to participate. Ten MRs were invited and all agreed to participate.

Both PAs and MRs were assessed at the Radboud University Medical Center, Institute for (Bio)Medical Education, The Netherlands. This study does not fall within the remit of the Medical Research Involving Human Subjects Act (WMO). All participation was voluntary, candidates signed a written informed consent, and all data were anonymized and handled strictly confidential.

### 2.2 Human patient simulator

An HPS is a patient simulator that reacts physiologically as if it was a real patient. Candidates were observed and evaluated as they pass through a simulated scenario like a real scenario. During the scenario that takes around 20 minutes, various competences as history taking, physical examination, diagnosing, treating, and discussion with the nurse were examined. In the current study, 2 scenarios with various phases and score lists were developed by the Radboud University Medical Center in the Netherlands. The research team was experienced with developing scenarios with score lists.^12^

The first scenario simulated acute respiratory distress syndrome (ARDS) and the second scenario simulated anaphylaxis, both in adult patients. Acute respiratory distress syndrome and anaphylaxis were chosen, because these diagnoses are common on ICUs, clinically relevant and suitable for developing score lists. Besides, we asked the candidates to handover the ARDS scenario to a colleague, who will take over the job. Detailed information about the content of the scenarios is presented in the addendum.

The performance of the candidates during the scenarios on the HPS was videorecorded and independently assessed by 2 intensivists. In total, 4 intensivists (JvO, MH, HK, and GvdN) were involved. The handover was scored independently by 2 teachers (GvdB and NB) with ICU experience as a nurse or PA. For each scenario, scenario‐specific checklists were developed to score the performance of the candidates. To increase the reliability of the assessment, the 4 intensivists first scored the performance of 2 candidates independently and discussed the score lists. Four items were changed in weight. Individual scores were expressed as percentages of the maximum score. The mean scores of the 2 intensivists were used in the data analysis. The inter‐rater reliability (intraclass correlation coefficient) among the intensivists was 0.91. This same procedure was done when scoring the handover by the teachers. The inter‐rater reliability (intraclass correlation coefficient) among the teachers was 0.78. The intensivists and teachers were blinded to the type of profession of the candidates (PA or MR). Most of the candidates had no experience with the HPS, only a few did use the HPS once during their training. None of the candidates were familiar with the HPS.

### 2.3 Data analysis

The following steps have been taken to analyse the data.

1. To determine the reliability of the OSCE, the standardized Cronbach alpha coefficient across the 7 phases (4 ARDS plus 3 anaphylaxis) was calculated.
2. Each participant completed 2 scenarios. Mean scores were calculated per individual participant for each separate scenario (ARDS and anaphylaxis).
3. The mean score for each separate scenario was calculated for PAs and MRs.
4. Since the data was normally distributed, we used the independent Student *t* test to investigate whether the mean scores of PAs were equal to that of MRs. We did that for the total of scenarios, for each scenario and for the 7 separate phases.

All analyses were conducted with the SPSS statistical software package version 20.0 for Windows (SPSS Inc, Chicago, IL).

## 3 RESULTS

### 3.1 Population

Of the 20 invited PAs, 11 participated in this study. In total, 10 MRs participated in the study. The mean age of PAs was 38 ± 7 years and 45% was male. They had on average 46 ± 17 months' work experience as a PA. In a university hospital, 36% was employed. The mean age of MRs was 27 ± 2 years and 40% was male. They had on average 12 ± 4 months' work experience as a MR. In a university hospital, 60% was employed.

### 3.2 Reliability

The overall Cronbach alpha of the test items (phases in the 2 scenarios) was 0.62. For PAs, the overall Cronbach alpha was 0.63 and 0.61 for MRs.

### 3.3 Level of clinical skills

**Figure 1:**
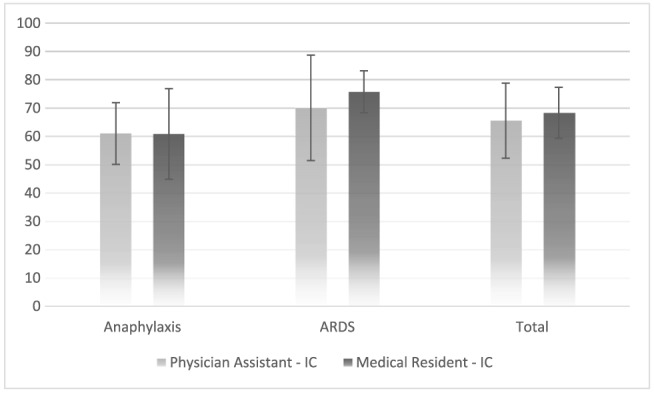
Mean scores in percentage (+/‐ standard deviation) of the performance of physician assistants (*n* = 11) and medical residents (*n* = 10) in the simulated ICU setting

The mean scores of the level of clinical skills of PAs and MRs are presented in Figure 1. The total mean score of PAs was 66% ± 13%, and the total mean score of MRs was 68% ± 9%. With respect to the separate scenarios: Mean scores on the scenario anaphylaxis were 70% ± 19% for PAs and 76% ± 7% for MRs. Mean scores on the scenario ARDS were 61% ± 11% for PAs and 61% ± 16% for MRs. There were no differences between PAs and MRs on the total score (*P* = .66), the different scenarios (ARDS, *P* = .85; anaphylaxis, *P* = .65) or the separate phases of the scenarios (*P* = .33‐.90).

## 4 DISCUSSION

This study shows that there is no significant difference in the level of clinical skills of PAs and MRs in the simulated ICU. Physician assistants and MRs score equally high on their performance in the different (phases of the) scenarios.

These results are consistent with our hypothesis and studies with the same design, performed in other specialities. As in our study, there was no difference in clinical competencies (ie, history taking, physical exam, communication, and report) between PAs and residents in anaesthesiology and surgery.^10^, ^11^ Furthermore, studies in the ICU reported the same quality of care by teams with PAs in comparison with teams without PAs.^8^, ^13^ In these studies, quality of care was measured by clinical indicators, for instance, mortality, fewer, pain or infections. The results of these studies could be in accordance with the outcomes in our study, as this possibly relates to the same topic.

We did not investigate the additional value of PAs in the ICU team. Other studies found a higher provider continuity on hospital wards on which PAs were employed than on wards with only MRs. The turnover of MRs is traditionally high due to use of recent medical graduates who are planning to do fellowship and the mandatory rotational cycles. Physician assistants generally do no rotate and constitute a factor of stability in the continually changing medical workforce at a hospital department.^9^ As a consequence, PAs might be more familiar with the clinical protocols and the procedures to, for example, request diagnostic tests and consultations of other specialities. Also, they know the routines of the nursing and medical team and could fill the gap between these teams.

In this study, we investigated whether PAs could be a safe substitute for MRs. Besides substitution of care, PAs could perform additional tasks that were normally the responsibility of the staff physician or resident, like integrating newly employed doctors, providing education, organizing nursing multidisciplinary consultation, taking part in medical emergency teams, or conducting quality projects.^5^, ^9^ We have not measured these topics in our study; however, we do believe that these topics are also relevant in ICUs.

### 4.1 Methodological considerations

The HPS is a unique method of evaluating and training the clinical skills and interprofessional communication skills of a team of different medical and nursing professionals. Originally, the HPS is not aimed as an assessment tool; however, since we did find a high inter‐rater reliability within intensivists in assessing the performance of the candidates, we were able to compare the performance of PAs with the performance of MRs. In this study, using the developed scenarios with score lists for the HPS as an assessment tool could be considered for PAs and MRs before entering the ICU. Although we were very happy with using the HPS as it reacts if it was a real patient, we also know the limitations. For instance, the skin of the patient did not change by an allergy. We do not know how PAs and MRs perform in direct clinical practice.

Furthermore, this is a small study with a limited amount of scenarios and 21 candidates.

We do not expect selection bias, since we included all MRs from 2 wards, one from a university hospital and one from a teaching hospital. Physician assistants were included by mailing. It could be that only PAs who scored high or low were included; however, we do not expect this since all PAs from several ICUs participated.

With respect to the generalizability, the goals of PA education are the same around the world, but the training methods are different.^6^ In the Netherlands, students spend nearly one‐third of their training time in one specialty, one‐third in various specialties (clinical rotations), and one‐third in classroom (integrated). Different ways of training could influence the level of clinical skills of PAs. We would recommend further research to investigate the level of clinical skills of PAs in ICUs across countries.

In conclusion, PAs in this study performed as well as MRs with 6 to 24 months experience in the ICU. This finding provides new evidence suggesting that PAs are safe to adjust to the ICU care team and supports a growing role for PAs in patient care management in the ICU setting. This has implications for medical/nursing directors, intensivists, hospitalist teams, and practice administrators in ICUs.

## ETHICS APPROVAL AND CONSENT TO PARTICIPATE

Not applicable. Informed consent was obtained from all candidates.

## CONSENT FOR PUBLICATION

Not applicable.

## AVAILABILITY OF DATA AND MATERIAL

The datasets used and/or analysed during the current study are available from the corresponding author on reasonable request.

## FUNDING

No funding.

## AUTHORS' CONTRIBUTIONS

All authors have been involved in the design of the study. A.v.V., G.v.B., J.v.O., and M.H. collected the data. G.v.B., J.v.O., and M.H. scored the performance of the candidates. A.v.V. analysed the data. The analyses and results were discussed with all authors (G.v.B., J.v.O., and M.H.). A.v.V. drafted the manuscript for submission to Critical Care. All authors have been involved in revising the manuscript. All authors read and approved the final manuscript.

## CONFLICT OF INTEREST

The authors declare no conflict of interest.

## ADDENDUM

### Scenario 1

An 18‐year‐old man, ideally bodyweight 75 kg, admitted to the ICU with a bilateral pneumonia developed ARDS. He had to be intubated and mechanical ventilation was started in a time‐cycled mode with initial high tidal volumes. The scenario is divided in 4 phases. In the first phase, points could be earned for recognition of high tidal volumes and adjustment of the ventilator to tidal volumes of 4 to 6 mL/kg. In the second phase, the patient developed hypoxia to less than 90% O2 saturation. Points could be earned for recognition, searching for explanations, examination of the patient, ordering an X‐ray of the chest, ordering bronchial aspiration and for adjustment of the ventilator. In the third phase, the patient developed severe hypoxia again, high pressure alarm on the ventilator, systolic blood pressure droppedto70 mm Hg and a silent left hemi‐thorax was present. Points could be earned for diagnosing a tension pneumothorax, for performing a needle decompression into the left second intercostal space in time and for ordering insertion of a chest tube. In the fourth phase, candidates had to handover the case to a colleague.

### Scenario 2

A 21‐year old man with a history of alcohol abuse is admitted to the ICU post‐operatively after an abdominal trauma due to stab wounds. Laparotomy revealed a perforation of the colon. Antibiotics were started. The patient is awake, detubated and breathing spontaneously, restless, and in pain. The scenario is divided in 3 phases. In the first phase, the patient is in pain. Points could be earned for assessment of a pain score, searching for explanations of pain, examination of the patient, ordering extra laboratory tests and treating pain with opioids, and re‐assess the pain‐score. In second phase, the patient developed a delirium. Points could be earned for recognition and treatment. In the third phase, the patient is in anaphylactic shock, has an inspiratory stridor, and develops itching of the skin. Points could be earned for recognition, creating a differential diagnosis, starting with oxygen, medication, ie, epinephrine, antihistamines and steroids, and stopping the antibiotics.
